# Technological and Sensory Quality of Gluten-Free Pasta Made from Flint Maize Cultivars

**DOI:** 10.3390/foods12142780

**Published:** 2023-07-21

**Authors:** Nicolás Francisco Bongianino, María Eugenia Steffolani, Claudio David Morales, Carlos Alberto Biasutti, Alberto Edel León

**Affiliations:** 1 Instituto de Ciencia y Tecnología de los Alimentos Córdoba (ICYTAC), National Scientific and Technical Research Council (CONICET), Universidad Nacional de Córdoba (UNC), Córdoba 5000, Argentina; nicolasbongianino@agro.unc.edu.ar (N.F.B.); eusteffolani@agro.unc.edu.ar (M.E.S.); david.morales@unc.edu.ar (C.D.M.); 2 Mejoramiento Genético Vegetal, Facultad de Ciencias Agropecuarias, Universidad Nacional de Córdoba (UNC), Casilla de Correo 509, Córdoba 5000, Argentina; biasutti@agro.unc.edu.ar; 3 Química Biológica, Facultad de Ciencias Agropecuarias, Universidad Nacional de Córdoba (UNC), Casilla de Correo 509, Córdoba 5000, Argentina

**Keywords:** gluten-free industry, maize quality, solvent retention capacity, Argentinian maize

## Abstract

The development of quality gluten-free products presents a major technological challenge in terms of structure, texture, and shelf life. However, there is insufficient information available to identify genotypes for obtaining gluten-free maize pasta of good acceptability and technological quality. The objective of this work was to evaluate the technological and sensory quality of gluten-free pasta made from different maize cultivars. The flint open-pollinated variety, flint inbred line, and three dent commercial hybrids were used. Grain and flour’s physical characteristics and chemical composition were determined. Gluten-free pasta was made via extrusion, and its quality traits were studied. A sensory evaluation test was carried out. Flint cultivars showed the lowest values on swelling index (both 1.77) and water absorption (124.30 and 134.58%). Pasta swelling index showed a negative association r = −0.77 to sodium carbonate retention capacity (*p* = 8.5 × 10^−5^) and water retention capacity (*p* = 6.6 × 10^−5^). Evaluators’ preference results showed a higher frequency of choices at the top level of preference (4) for the flint open-pollinated variety C6006. Thus, evaluators’ choices showed a positive association between sample preference and firmness. Pasta preference and technological quality have a direct relationship with fast tests over grain, such as test weight and float index.

## 1. Introduction

Coeliac disease (CD) is an intestinal condition in genetically predisposed patients caused by the ingestion of foods containing gluten. Gluten is a combination of proteins called gliadins and glutenins. Additionally, this protein matrix has unique characteristics that promote its use in many food products, such as extensibility, resistance to stretching, tolerance to mixing, and gas retention capacity [1].

Marti and Pagani [2] claimed that the gluten network of dry pasta is distributed around the starch granules relatively evenly and regularly. Furthermore, this network affects the starch swelling capacity and the cooking quality of the pasta, expressed as stickiness, cooking loss, water absorption, and firmness [3,4].

The elimination of gluten results in many gluten-free products available on the market having undesirable characteristics, such as low quality and poor mouthfeel and taste. For this reason, there is a growing trend to investigate the application of alternative grains as a healthy option with the potential for gluten-free products [5]. On the other hand, there is currently a major technological challenge for the development of quality gluten-free products due to the absence of gluten’s key role in terms of structure, texture, and shelf life. In addition, their nutritional value is often below the nutritional standard [6].

Maize has a high nutritional impact worldwide because it provides macronutrients, phenolic compounds, phytosterols, and carotenoids. Hence, products derived from dry-milled maize are used in gastronomy to prepare regional foods like polenta [7]. Also, maize flour has some advantages, such as a yellow colour, a bland taste, and easy digestion [8]. For this reason, as maize is gluten-free, it is considered a safe raw material for coeliacs and is used in the production of gluten-free pasta. Thus, it is necessary to replace the gluten network with a new and efficient organization of starch when this is the only material used in the production of this food [9]. 

Gluten-free noodles can be easily transported from their processing centre and have a long shelf life. Despite these advantages, it is necessary to continue improving the quality characteristics and solve technological problems in producing maize noodles [10]. In this regard, Scarton and Clerici [11] mentioned that maize pasta, like all gluten-free pasta, is more brittle, tends to lose more material during cooking, and is less attractive to the consumer. All of these defects are due to the lack of gluten, which can form a continuous matrix around the starch granules.

On the other hand, the selection of raw materials for gluten-free pasta production usually does not take into account the starch characteristics and only focuses on checking the gluten absence. In addition, heat treatments or additives are used in the formulation to improve the cooking behaviour and GF technology of the product. In any case, after gelatinization, the starch macromolecules must reorganize into new structures, which are capable of retarding swelling and leaching during cooking [2].

At the same time, texture-related properties are the main factor in evaluating pasta quality. Thus, quality pasta should exhibit high firmness and non-stickiness, and on the other hand, low cooking loss, which can be specifically attributed to the specific structural compound organization of starch and protein [12]. The improvement in gluten-free pasta has focused on the selection of raw materials for formulations and processing method variation [13]. 

Argentina is one of the world’s leading producers of maize and the only exporter of flint maize, which is non-genetically modified. It is also well known for its good dry milling performance and the quality of the final products, such as breakfast cereals, snacks, and other textured ingredients [14]. On the other hand, the introduction of the dent germplasm has achieved yield increases of 10–20% in the field. This has led to the massive adoption of dentate and semi-dentate genotypes, leaving flints behind [15]. Some authors mentioned that dent genotypes are characterised by a lower proportion of vitreous components in their endosperm and a lower grain formation efficiency. On the other hand, these materials have a greater number of grains per plant and a higher grain weight, which result in a higher yield. This behaviour is associated with traits during the flowering period, such as a higher rate of plant biomass accumulation, higher plant growth per grain, and a greater distribution of plant biomass in the ear [14]. 

The trend to select germplasms with higher yield potential has indirectly modified the traditional grain composition and hardness of Argentine maize. Current hybrids on the market are less suitable for dry milling than traditional flint, showing more floating kernels and lower hectoliter weight and crystalline fraction [16]. Also, Ortiz-Monasterio et al. [17] indicated that breeding strategies should aim to generate maize cultivars that have acceptable end-use quality. Thus, the likelihood that these new cultivars will be adopted by farmers and that foods made from them will be accepted increases. For this reason, it is essential to highlight that insufficient information is available to identify genotypes for obtaining gluten-free maize paste with good acceptability and technological quality. The objective of this work was to evaluate the technological and sensory quality of gluten-free pasta made from different maize cultivars. 

## 2. Materials and Methods

### 2.1. Genetic Material

The genotypes used were orange flint open-pollinated variety (OPV) “C6006” and yellow flint inbred line (IL) “CIM06”, both developed in the Plant Breeding Department of the Faculty of Agricultural Sciences, National University of Cordoba (FCA–UNC), as well as three yellow dent commercial hybrids, (H) “P1815”, “P2089”, and “AX882” used as control. The late sowing was carried out in mid-December 2020 at the FCA-UNC School Field (31°29′ S; 64°00′ W), and two replicates were planted. 

### 2.2. Physical Determinations

The weight of 100 grains was determined with an analytical balance (W100) following the AACC Method 56-35.01. The test weight (TW) was estimated using a Schopper scale (AACC Method 55-10) (AACC Approved Methods of Analysis, 2010). The flotation index used as a grain hardness indicator was estimated following the Palacios-Rojas [18] method, with a sodium nitrate solution (density (q) 1250 ± 0.001).

### 2.3. Flour Physical and Chemical Characterization

Wholegrain samples were ground in duplicate using a cyclone mill (Cyclotec CT193, Foss, Hillerod, Denmark), and wholegrain flours with particles < 1000 μm were obtained. Samples were stored at 4 °C until their respective determinations, which were made in duplicate. The macronutrient contents (protein, lipids, ash, and starch) were determined according to 46-10.01, 30-25.01, 08-01.01, and 76-13.01 AACC methods, respectively. The commercial kit (Megazyme Ireland International, Ltd., Bray, Ireland) was used for the amylose/amylopectin content determination. All determinations were expressed in g per 100 g of sample on a dry basis. The particle size was obtained via optical microscopy, and analysis of the particle size distribution was recorded for 90% or more of the sample in µm (D90). A laser particle size analyser (HORIBA LA 960, Kyoto, Japan) was used.

### 2.4. Solvent Retention Capacity

The solvent retention capacity of flours was evaluated according to Cappa et al. [19] with some minor modifications. Thus, 1.5 g of the sample was weighed in a 15 mL centrifuge tube. Then, 7.5 mL of each solvent—water and 5.0% (*w*/*w*) sodium carbonate in water— was added separately to the samples, and the mixtures were vigorously shaken for 5 s. The mixtures were then shaken every 5 min for 20 min and centrifuged for 15 min at 2000× *g* (Sorvall ST 40R, Thermo Fisher Scientific, Bremen, Germany) at room temperature (25 °C). The solvent retention capacity (SRC) was calculated as the weight of solvent held by samples after centrifugation, supernatant separation, and gel drainage for 10 min, and expressed as a percentage of sample weight, based on a base humidity of 14%.

### 2.5. Pasta Making 

GF pasta was made using wholegrain flour 94 g, pasteurised food-grade egg albumin 2 g (Todo Droga, Córdoba, Argentina), pregelatinized maize starch 3 g (Ingredion, Buenos Aires, Argentina), salt 1 g, and water. Fettuccine pieces 20 cm in length were obtained through conventional cold extrusion (30–40 °C) (Dolly, Imperia & Monferrina S. P. A., Castell’Alfero, Asti, Italy) [20]. The pasta was dried at 40 °C in an air convection drier for 240 min. The samples were stored in airtight containers at room temperature until needed.

### 2.6. Pasta Quality Parameters

The pasta was cooked until its optimal cooking time. This was obtained by cooking 5 g of pasta in 200 mL of boiling distilled water and recording when the central white region disappeared [21]. An Instron universal texture machine (Instron, High Wycombe, UK) equipped with a 500 N cell was used. Four replicates for each sample were analysed. Water absorption (%), cooking loss (%), and swelling index were determined. Firmness (N), adhesiveness (J), springiness, and gumminess (N) were calculated from curves obtained after two compressions of 50% of a double pasta strand. All determinations were performed according to Bustos et al. [22] with some modifications.

### 2.7. Sensory Analysis

The sensory profiling was performed by a semi-trained panel of twenty-seven individuals (ten males and seventeen females) aged between 23 and 60 years, according to IRAM, Standards 20002: 1995, 20010: 1997, and 20014: 1998. Instructions with definitions of sensory attributes (Table 1) and three samples (one commercial hybrid, one inbred line, and one OPV) were presented to each evaluator at the same time. Considering what was mentioned by the authors [16], the genotypes selected for the sensory analysis were the two flint materials developed at the FCA-UNC to represent the traditional Argentinean maize cultivars and the commercial control hybrid that showed the most significant differences in terms of OPV and IL. The samples were identified with four arbitrary numbers. A registration and evaluation form was given to each judge. Discontinuous bipolar 7-point scales were used, where 1 represented the lowest intensity and 7 the highest intensity of a particular attribute. The pasta was served cooked in 200 mL thermally expanded polystyrene cups. Drinking water was provided for rinsing and palate cleansing between each sampling unit. To know the acceptability of the pasta, a ranking test was conducted between 1 (least accepted sample) and 4 (most accepted sample) [23]. 

### 2.8. Statistical Analysis

Statistical analyses were carried out using InfoStat statistical software (InfoStat, Cordoba, Argentina, Version 2020). Variable relationships were established using Pearson’s correlation test with a significance level of *p* < 0.05 and 0.01. An analysis of variance was also performed with the LSD Fisher comparison test and a significance level of *p* < 0.05. Also, a Principal Component Analysis was used for sensory panellist evaluations.

## 3. Results and Discussion 

### 3.1. Grain and Flour Physical Traits 

The genotypes showed grain and flour physical traits and chemical composition statistical differences (Table 2). The smallest P100 expression was obtained for hybrid P1815. Also, the OPV and IL showed the highest values for test weight and lower flotation index. On the other hand, flour particle size showed a bimodal distribution (Table 2), with IL having the highest value in both cases (92.69 µm for D901 and 823.42 µm for D902). Endosperm hardness affects maize fragmentation and particle size. Therefore, maize with a soft endosperm easily breaks into small particles during crushing. At the same time, the endosperm of yellow maize (C6006 and CIM06) has a higher hardness due to the high number of internal protein matrices [24]. The authors in [25] concluded that maize kernel density was the best predictor of dry milling yields because flaked grit yields were significantly increased by selecting maize with low susceptibility to breakage and a high hectoliter weight. In addition, some results reported that kernel hardness showed a significant correlation with protein content, test weight, and kernel density. Thus, flint maize is in high demand because of its high milling yields of large endosperm grits, as well as the particular quality that it provides to a wide variety of end-use products, such as corn flakes, snacks, and other textured ingredients [15]. 

### 3.2. Solvent Retention Capacity and Flour Chemical Composition

The flours from flint genotypes showed more water retention than commercial hybrids, with values between 255.77 and 256.31%. In addition, OPV showed the highest sodium carbonate absorption capacity in its flour, with a value of 265.10% (Table 2). Damaged starch content showed significant differences between genotypes, where flint IL had the highest value (26.1%). Shi et al. [26] observed that the smaller the flour particle size, the higher the hydration capacity (water-retaining capacity) of ground maize flour during the grinding process. Also, the polysaccharide chains form a porous matrix structure, which is related to the hydration properties as they can retain large amounts of water through hydrogen bonds. In addition, León et al. [27] showed that the level of damaged starch varies with the severity of grinding and the kernel hardness. Thus, milling hard-textured grains generates a higher number of physically damaged starch granules as they require more energy than soft-textured grains in this process. In addition, the altered surface of the damaged starch granule generates a hydrophilic bond increase, thus increasing the flour water absorption capacity [28]. Significant correlations found in our work agree with the findings just described: particle size (D901 and D902) /damaged starch r = 0.58 (*p* = 0.0078) and 0.67 (*p* = 0.0013). Also, these positive relationships (r coefficient) were expressed for particle size and solvent retention capacity D901/ScRC and WRC = 0.52 and 0.54 (*p* = 0.0192 and 0.0136), and D902/WRC = 0.54 (*p* = 0.0137).

Similar values were observed by the authors when dent grains had lower TW and protein content and higher starch percentage than flint maize. Thus, kernel hardness has traditionally been related to kernel protein concentration, especially to the content of some specific endosperm proteins called zeins [29]. In addition, Robutti et al. [30] found comparable values for amylose content (20.9–22.8%) in different maize cultivars with variable grain texture. The genotypes C6006 and CIM06 showed the highest protein content (10.33 and 10.47%), and the IL had the lowest starch percentage (63.96%). The hybrid P1815 had less amylose content than other cultivars (Table 2). 

### 3.3. Pasta Quality Traits

Statistically significant differences were found for pasta-cooking features (Table 3). Flint cultivars showed the lowest values in both swelling index (both 1.77) and water absorption (124.30 and 134.58%). It has been reported that wheat spaghetti with low protein content absorbs more water than high-protein-content spaghetti. This could be attributed to water diffusion into the starch granules being impeded by a strong protein network [31].

Pearson correlation (*p* < 0.05 and 0.01) results were obtained between grain physical and pasta quality traits. The float index showed a positive relationship with SI (r = 0.86 and *p* = 1.2 × 10^−6^), WA (r = 0.84 and *p* = 3.3 × 10^−6^) and a negative one with pasta firmness (r = −0.78 and *p* = 4.4 × 10^−5^). On the other hand, TW was negatively associated with water absorption (r = −0.71 and *p* = 4.5 × 10^−4^) and swelling index (r = −0.72 and *p* = 3.2 × 10^−4^). In addition, W100 showed a negative association with GS (r = −0.53 and *p* = 0.02) and FS (r = −0.48 and *p* = 0.03).

Regarding cooking loss (CL), all the pasta showed similar values, around 7%. Therefore, the pasta performed well in this parameter as these CL values were lower than the 12% limit suggested for good-quality gluten-free pasta [32]. This behaviour can be related to that mentioned by Schoenlechner et al. [33], who mentioned that different factors can improve cooking losses. One of these factors is ensuring the correct dough moisture content because, if it is too high, the dough sticks to the machinery and is challenging to work with. On the other hand, adding external protein sources such as egg white improves the dough in terms of firmness and cooking loss. The flint genotypes showed more firmness than hybrid cultivars with 21.65 and 19.68 N values (Table 3). Also, a positive association was found between ScRC/WRC and pasta firmness (*p* < 0.05), with an r coefficient of 0.45 and 0.52, respectively. The authors of [34] found similar firmness results for maize pasta (21.1 N). Furthermore, they explained that during cooking, loosening of the compact dough structure and leaching of materials occur due to the presence of water-soluble components and alterations in the protein–starch matrix [35]. In addition, the development of the pasta’s texture and stickiness depends on the starch’s state and surface structure. Thus, firm texture, low adhesiveness, and low cooking loss are more important for good-quality pasta [36]. 

Moreover, D901 and D902 correlated negatively with SI (r = −0.64 and −0.57, *p* = 0.0023 and 0.0087), WA (r = −0.64 both, *p* = 0.022 and 0.0026), and D902/AS (r = −0.51, *p* = 0.0229). Some authors [37] mentioned that during the pasta-cooking process, molecular changes occur in the structure of starch and proteins. This could affect water absorption and pasta characteristics, such as final weight and firmness, whereby higher water absorption results in higher cooked pasta weight and lower firmness. In addition, regarding water absorption, Bresciani et al. [38] mentioned that the value decreased because the highly packed structure of starch limited both gelatinization during the pasta-making process and, therefore, pasta water absorption during cooking as well.

Pasta swelling index showed a negative association r = −0.77 with ScRC (*p* = 8.5 × 10^−5^) and WRC (*p* = 6.6 × 10^−5^). On the other hand, pasta water absorption showed r = −0.76 to ScRC (*p* = 1.1 × 10^−4^) and r = −0.77 to WRC (*p* = 6.1 × 10^−5^), probably because starch granules swell to the maximum in the presence of hot water and because there was a higher content of damaged starch, and then, the solubility of leached compounds increases. Furthermore, these conditions favour the formation of amylose networks, which inhibit the swelling of starch granules [39]. For their part, Batterman-Azcona and Hamaker [30,40] mentioned that extrusion alters the structure and composition of the original endosperm matrix because it generates a disruption of zein bodies and the release of proteins during extrusion. This process contributes to the formation of disulfide-bound zein polymers during the cooking of maize flour in boiling water. Thus, the degree of aggregation of zeins is a determinant of the sensory and textural properties of extruded maize products. Barrera et al. [41] stated that the damaged starch granules in baked products typically release part of the absorbed water.

### 3.4. Sensory Evaluation

**Figure 1:**
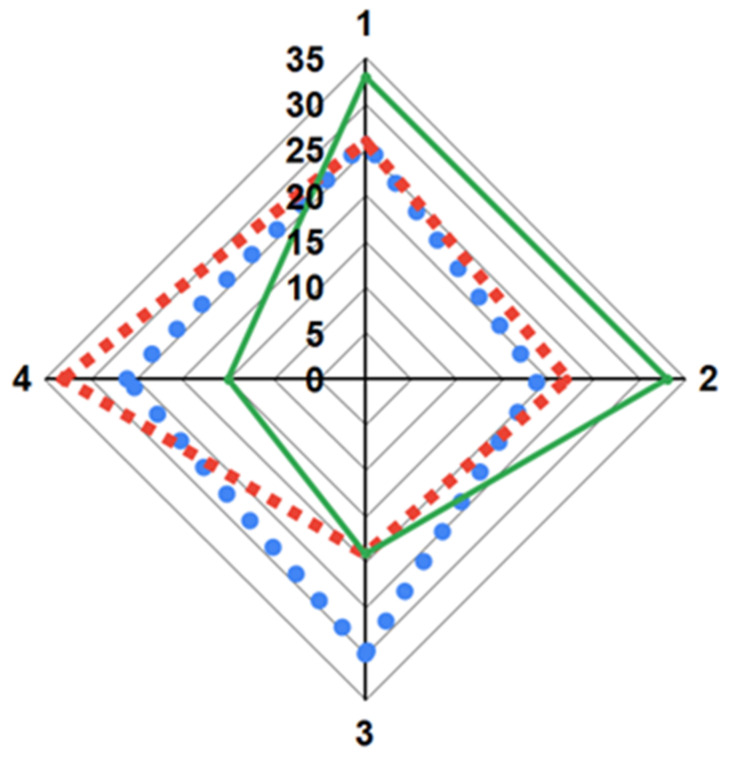
Frequency of evaluators’ preference choices. Highest preference level = 4. AX882 (● Blue), C6006 (■ Red) and CIM06 (▬ Green).

**Figure 2:**
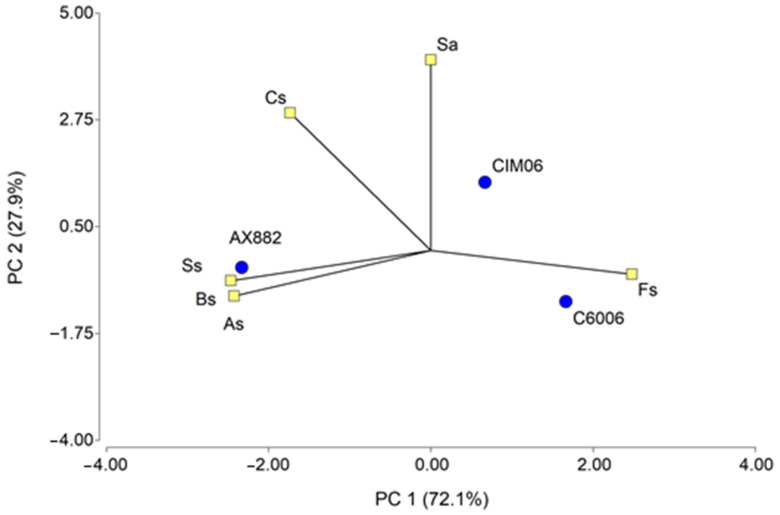
Principal component analysis for sensory parameters. As, Adhesiveness; Fs, Firmness; Bs, Brightness; Cs, Chewiness; Ss, Springiness and Sa, Surface appearance.

The results of the panellists’ preferences (Figure 1) showed a higher frequency of choices at the highest level of preference (4) for OPV C6006. On the other hand, principal component analyses explained 72.1% of PC1 and 27.9% of PC2 of the total variability in the sensory descriptors (Table 4). Thus, the panellists’ choices showed a positive association between the most preferred sample (C6006) and firmness (Figure 2). These results were similar to those reported by the authors of [37], who confirmed a better paste quality in pieces with high firmness. Furthermore, OPV was characterized by low values for Ss, Bs, and As attributes. This behaviour in adhesiveness is related to a lower exudation of starch granules from the paste matrix to its surface during firing, which results in lower adhesiveness [42]. On the other hand, the pasta obtained from the commercial hybrid AX882 showed the highest scores in terms of adhesiveness, gloss, and elasticity versus firmness, with low scores over the other two materials. Considering the variability explained by PC2, we can highlight that the attribute with the greatest impact on genotype differentiation is Sa. Thus, the material with a higher preference C6006 was associated with a lower expression of this variable. On the other hand, the genotype CIM06, which was similar to the most preferred sample in all other attributes, showed the highest value for surface appearance (Table S1). Likewise, Martínez et al. [23], showed that the panellists had a greater preference for pasta with fewer dots on the surface of the food, in other words, with a better surface appearance.

## 4. Conclusions

In this study, we found that the preference of the evaluators and the technological quality of the pasta have a direct relationship with rapid grain tests, such as test weight and flotation index. On the other hand, we found that hard-grained and high-hectoliter-weight genotypes generate flours with high solvent retention due to the higher content of damaged starch, and after extrusion and cooking, the pasta obtained shows low water retention and absorption and higher firmness. The information showed that direct and indirect relationships between variables can be used by the industry when selecting suitable raw materials for the production of gluten-free maize pasta. Genotype C6006 showed the highest potential for gluten-free pasta production since it is represented by pasta characteristics, such as high firmness, low stickiness, and surface appearance, and finally, the highest preference of the panellists. Also, the OPV can be used in genetic improvement programmes to generate new genotypes for good-quality pasta production.

## Figures and Tables

**Table 1:** Definition of attributes used in the sensory analysis of gluten-free pasta.

Attribute	Instructions
Brightness	Take a strand of pasta and place it in line with your view, turn it slightly so that the light falls on it, and see how much it reflects.
Surface appearance	Take a strand of pasta and place it on the plate. Observe and count how many points you can observe.
Firmness	Take one strand of pasta at a time, place it between the incisors, and bite evenly, assessing the force required to compress and cut evenly.
Chewiness	Take a strand of pasta, place it between the molars, and chew it at a steady, speed. Count the number of times you chew to reduce it to a swallowable state.
Springiness	Take a strand of pasta from one of its ends and roll it out. Assess the degree to which the pasta reaches its original length/stretch resistance without breaking.
Adhesiveness	Place the pasta in the mouth, pressing it against the roof of the mouth, and determine the force required to remove it with the tongue.

**Table 2:** Grain and flour physical traits and chemical composition average values.

Genotype	AX882	P1815	P2089	C6006	CIM06
P100 (g)	35.27 ± 3.94 ^bc^	31.25 ± 4.53 ^a^	37.98 ± 0.38 ^c^	32.95 ± 1.17 ^ab^	33.40 ± 1.16 ^ab^
TW (Kg/HL)	85.00 ± 1.92 ^b^	88.80 ± 1.17 ^c^	81.60 ± 1.03 ^a^	92.00 ± 0.73 ^d^	92.50 ± 1.10 ^d^
FI (%)	90.50 ± 3.42 ^d^	74.00 ± 6.32 ^c^	94.00 ± 1.63 ^d^	21.50 ± 5.51 ^b^	10.50 ± 4.12 ^a^
WRC (%)	237.44 ± 2.06 ^a^	234.46 ± 6.07 ^a^	234.33 ± 8.94 ^a^	256.31 ± 9.39 ^b^	255.77 ± 10.15 ^b^
ScRC (%)	213.19 ± 29.9 ^a^	233.21 ± 2.27 ^ab^	224.19 ± 12.97 ^a^	263.10 ± 6.29 ^c^	245.97 ± 2.41 ^bc^
D901 (µm)	78.35 ± 3.56 ^abc^	70.75 ± 12.10 ^a^	72.94 ± 14.79 ^ab^	91.47 ± 13.77 ^bc^	92.69 ± 13.61 ^c^
D902 (µm)	631.86 ± 100.05 ^ab^	693.93 ± 187.04 ^abc^	524.39 ± 143.21 ^a^	722.87 ± 77.71 ^bc^	823.42 ± 53.96 ^c^
Ash (%)	1.54 ± 0.04 ^ab^	1.66 ± 0.10 ^c^	1.52 ± 0.04 ^a^	1.73 ± 0.13 ^c^	1.65 ± 0.06 ^bc^
Protein (%)	8.46 ± 0.53 ^b^	7.95 ± 0.25 ^b^	7.39 ± 0.27 ^a^	10.33 ± 0.58 ^c^	10.47 ± 0.21 ^c^
Oil (%)	5.15 ± 0.33 ^a^	6.05 ± 1.65 ^b^	6.11 ± 1.67 ^b^	4.67 ± 0.16 ^a^	6.77 ± 0.12 ^b^
Starch (%)	74.00 ± 4.74 ^d^	72.96 ± 1.86 ^cd^	65.93 ± 2.99 ^ab^	68.98 ± 2.59 ^bc^	63.96 ± 2.07 ^a^
Amylose (%)	22.08 ± 1.29 ^b^	18.27 ± 3.94 ^a^	21.33 ± 0.61 ^b^	22.04 ± 1.15 ^b^	21.83 ± 0.57 ^b^
Amylose/Starch (%)	0.30 ± 0.03 ^ab^	0.25 ± 0.06 ^a^	0.32 ± 0.01 ^b^	0.32 ± 0.02 ^b^	0.34 ± 0.02 ^b^
Dam.S (%)	14.29 ± 1.97 ^a^	20.83 ± 0.70 ^b^	13.61 ± 0.75 ^a^	22.92 ± 3.05 ^c^	26.1 ± 0.71 ^d^

Average values with different letters indicate statistical differences (LSD Fisher, *p* < 0.05). P100, hundred-grain weight; TW, hectolitre weight; FI, float index; Dam.S, Damaged starch content; WRC, water retention capacity; ScRC, sodium carbonate retention capacity; D901, particle size represented by 90% or more of the total particles on first distribution; D902, particle size represented by 90% or more of the total particles on second distribution. ± Standard deviation.

**Table 3:** Pasta quality traits.

Genotype	SS	GS (N)	FS (N)	AS (J)	SI	WA (%)	CL (%)
AX882	0.89 ± 0.03 ^b^	5.53 ± 1.37 ^a^	13.95 ± 2.31 ^a^	0.00021 ± 0.000054 ^a^	2.28 ± 0.18 ^c^	296.10 ± 54.55 ^c^	7.63 ± 0.76 ^a^
P1815	0.82 ± 0.07 ^a^	8.03 ± 2.87 ^c^	17.56 ± 2.57 ^b^	0.00026 ± 0.000042 ^ab^	2.06 ± 0.08 ^b^	216.50 ± 42.90 ^b^	7.30 ± 0.64 ^a^
P2089	0.83 ± 0.07 ^a^	7.44 ± 0.93 ^bc^	17.09 ± 1.01 ^b^	0.00031 ± 0.000027 ^b^	2.22 ± 0.12 ^bc^	278.86 ± 51.22 ^c^	6.78 ± 0.75 ^a^
C6006	0.83 ± 0.05 ^ab^	6.66 ± 0.79 ^abc^	19.68 ± 1.83 ^c^	0.00023 ± 0.000046 ^a^	1.77 ± 0.14 ^a^	124.30 ± 44.98 ^a^	6.98 ± 0.89 ^a^
CIM06	0.82 ± 0.08 ^a^	6.56 ± 0.96 ^ab^	21.65 ± 1.31 ^c^	0.00022 ± 0.00001 ^a^	1.77 ± 0.10 ^a^	134.58 ± 24.27 ^a^	7.10 ± 1.25 ^a^

Average values with different letters indicate statistical differences (LSD Fisher, *p* < 0.05). SS, springiness; GS, gumminess; FS, firmness; AS, adhesiveness; SI, swelling index; WA, water absorption; CL, cooking loss. ± Standard deviation.

**Table 4:** Eigenvalues from principal component analysis.

Variable	PC1	PC2
Value	4.32	1.68
Proportion	0.72	0.28
Cumulative proportion	0.72	1
Sensory parameters		
Bs	−0.47	−0.19
Sa	1.20 × 10^−4^	0.77
Fs	0.48	−0.1
Cs	−0.33	0.56
As	−0.47	−0.19
Ss	−0.47	−0.12

As, Adhesiveness; Fs, Firmness; Bs, Brightness; Cs, Chewiness; Ss, Springiness and Sa, Surface appearance.

## Data Availability

The data presented in this study are available on request from the corresponding author.

## Supplementary Materials

The following supporting information can be downloaded at: https://www.mdpi.com/article/10.3390/foods12142780/s1, Table S1: Relative frequency values for sensory evaluation traits.
