# Supplementary material for: Technological and Sensory Quality of Gluten-Free Pasta Made from Flint Maize Cultivars
Source: Foods. 2023 Jul 21;12(14):2780. doi: 10.3390/foods12142780 (PMC10378873; doi:10.3390/foods12142780)
Supplement: Supplementary file 1 [file foods-12-02780-s001.zip › foods-2491101-supplementary.pdf]

Table S1. Relative frequency values for sensory evaluation traits.

Genotype	Trait	1	2	3	4	5	6	7
AX882	Bs	15	11	30	0	30	7	7
	Cs	4	30	33	0	11	19	4
	Ss	19	19	37	0	15	4	7
	As	30	19	19	0	11	19	4
	Sa	11	26	33	15	0	11	4
	Fs	15	41	19	15	7	4	0
C6006	Bs	19	26	0	15	0	26	15
	Cs	7	37	26	0	15	11	4
	Ss	33	30	15	11	4	4	4
	As	41	26	0	0	19	7	7
	Sa	7	44	22	0	11	11	4
	Fs	15	26	22	22	0	15	0
CIM06	Bs	22	22	26	11	11	4	4
	Cs	15	26	19	15	19	4	4
	Ss	26	33	22	0	4	11	4
	As	26	33	0	22	0	4	15
	Sa	11	7	15	0	26	30	11
	Fs	26	15	30	11	7	7	4

Relative frequencies in percentages for the 7-point measuring scale. Bs, Brightness; Cs, Chewiness; Ss, Springiness; As, Adhesiveness; Sa, Surface appearance and Fs, Firmness.
